# Successful treatment of nivolumab and ipilimumab triggered type 1 diabetes by using sodium-glucose transporter 2 inhibitor: a case report and systematic review

**DOI:** 10.3389/fpubh.2023.1264056

**Published:** 2023-12-01

**Authors:** Makoto Fujiwara, Masaru Shimizu, Tatsuya Okano, Yuko Maejima, Kenju Shimomura

**Affiliations:** ^1^Department of Bioregulation and Pharmacological Medicine, Fukushima Medical University School of Medicine, Fukushima, Japan; ^2^Department of Diabetes, Endocrinology and Metabolism, Tsukuba Medical Center, Ibaraki, Japan; ^3^Department of Neurology, Matsumura General Hospital, Fukushima, Japan

**Keywords:** nivolumab, ipilimumab, diabetes, SGLT2 inhibitor, insulin

## Abstract

**Objective:**

Checkpoint inhibitors (CPIs) can trigger complications related to the autoimmune process such as CPI-triggered diabetes mellitus. The typical treatment for CPI-triggered diabetes is insulin, but a detailed therapeutic method has not yet been established. To prevent severe symptoms and mortality of diabetic ketoacidosis in advanced-stage cancer patients, the establishment of effective treatment of CPI-triggered diabetes, other than insulin therapy, is required.

**Methods:**

We present a case of a 76-year-old man with CPI-triggered diabetes who was treated with nivolumab and ipilimumab for lung cancer. We also conducted a systematic review of 48 case reports of type 1 diabetes associated with nivolumab and ipilimumab therapy before June 2023.

**Results:**

The patient’s hyperglycemia was not sufficiently controlled by insulin therapy, and after the remission of ketoacidosis, the addition of a sodium-glucose transporter (SGLT) 2 inhibitor, dapagliflozin, improved glycemic control. Most of the reported nivolumab/ipilimumab-induced type 1 diabetes was treatable with insulin, but very few cases required additional oral anti-diabetic agents to obtain good glucose control.

**Conclusion:**

Although SGLT2 inhibitors have been reported to have adverse effects on ketoacidosis, recent studies indicate that the occurrence of ketoacidosis is relatively rare. Considering the pathological mechanism of CPI-triggered diabetes, SGLT2 inhibitors could be an effective choice if they are administered while carefully monitoring the patient’s ketoacidosis.

## Introduction

The development of checkpoint inhibitors (CPIs) was a major breakthrough for the treatment of various cancers, including advanced-stage cancers that were previously considered untreatable (1, 2). These drugs restore a deficient anti-cancer immune response by blocking cytotoxic T-lymphocyte 4 (CTLA-4) or programmed cell death 1 (PD-1) receptor and its ligand PDL-1 (3–5). The introduction of CPIs was a paradigm shift in cancer treatment. While these new effective treatments are widely used, the number of CPI-triggered adverse events is increasing.

CPI-triggered adverse events tend to target endocrine organs such as hypophysis, thyroid, and insulin-secreting islets(6, 7).

When islets are affected in CPI-triggered adverse events, a patient presents features similar to those of type 1 diabetes(7, 8). CPI-triggered diabetes is rare (approximately 1%) but potentially life-threatening(7, 9–11). Therefore, it requires effective and reliable treatment. In most cases, CPI-triggered diabetes is treated with insulin injections as suggested by the guidelines(12, 13). However, there are no suggestions for the treatment when insulin therapy cannot achieve good glycemic control.

In type 1 diabetes, in addition to insulin therapy, the use of sodium-glucose transporter (SGLT) 2 inhibitors is suggested as an additional treatment in Japan. A recent Dapagliflozin Evaluation in Patients with Inadequately Controlled Type 1 Diabetes (DEPICT) clinical trial showed the beneficial effect of using dapagliflozin in patients with type 1 diabetes inadequately controlled by insulin (14, 15). In 2019, the Japanese Ministry of Health, Labour and Welfare approved dapagliflozin as an oral adjunct treatment to insulin for patients with type 1 diabetes. SGLT2 inhibitors are known to have a low risk of developing ketoacidosis, but the risk is considered to be higher than that of other anti-diabetic agents (16, 17). Because CPI-triggered diabetes may present with ketoacidosis, SGLT2 inhibitors are not considered a treatment option, and insulin treatment is the primary treatment. However, because type 1 diabetes and CPI-triggered diabetes are both insulin-deficient diseases, the addition of SGLT2 inhibitors on top of insulin therapy may provide better glycemic control in CPI-triggered diabetes if they are used with caution.

Here, we report a case of successful glycemic control by adding an SGLT2 inhibitor on top of insulin therapy in a CPI-triggered diabetes patient. The addition of SGLT2 inhibitors on top of insulin therapy significantly improved the glucose level. The results in the present case indicate the possible use of SGLT2 inhibitors for the treatment of CPI-triggered diabetes. In addition, we conducted a systematic review of published case reports. This systematic review aims to provide a comprehensive evaluation of treatment options for CPI-triggered diabetes and shed light on potential therapeutic strategies.

## Case report

A 76-year-old Japanese man under treatment of PD-1 inhibitor (nivolumab) and CTLA-4 inhibitor (ipilimumab) for lung cancer, for 3 months, presented with casual blood glucose 574 mg/dL and HbA1c 7.7%. The patient had no history of diabetes, and this marked the initial onset of hyperglycemia. His primary cancer, situated in the left mediastinum, as well as his supraclavicular lymph node metastasis, exhibited signs of regression due to the therapeutic intervention. However, multiple metastatic cancers were observed in both lungs. No hepatic or adrenal metastasis was observed. No ascites or pleural effusion was noted.

The anti-GAD antibody was negative. RBC count was 427 × 10^4^/μL, creatinine level was 1.12 mg/dL, eGFR was 49 mL/min/1.73m^2^, and potassium level was 5.2 mmoL/L. Other laboratory data are provided in Table 1.

**Table 1:** Laboratory data.

Factor	Result	Reference range
Total protein (g/dL)	7.6	6.1–8.1
Albumin (g/dL)	4.6	4.1–5.1
BUN (mg/dL)	33.1	8.0–20.0
Creatinine (mg/dL)	1.12	0.65–1.07
Sodium (mmol/L)	137	138–145
Chloride (mmol/L)	96	101–108
Potassium (mmol/L)	5.2	3.6–4.8
Amylase (U/L)	65	44–132
ALP (U/L)	116	38–113
AST (U/L)	17	13–30
ALT (U/L)	21	10–42
LD (U/L)	160	124–222
CK (U/L)	75	59–248
Total bilirubin (mg/dL)	0.6	0.4–1.5
Plasma glucose (mg/dL)	574	73–109
CRP (mg/dL)	1.11	< 0.14
White blood cell (X10^2^/μL)	145	33–86
Red blood cell (X10^4^/μL)	427	435–555
Hb (g/dL)	13.8	13.7–16.8
Hct (%)	40.1	40.7–50.1
MCV (fl)	93.9	83.6–98.2
MCH (pg)	32.3	27.5–33.2
MCHC (g/dL)	34.4	31.7–35.3
Platelet (X10^4^/μL)	32.2	15.8–34.8
Neutrophil (%)	84.1	38.5–80.5
Lymphocyte (%)	14.4	16.5–49.5
Monocyte (%)	1.2	2.0–10.0
Eosinophil (%)	0.1	0.0–8.5
Basocyte (%)	0.2	0.0–2.5

The patient was diagnosed with fulminant type 1 diabetes provoked by the CPIs. His pre-prandial blood glucose levels ranged between 358 and 544 mg/dL, and insulin glargine before bed (0-0-0-6 U) plus insulin (Humulin R) sliding scale therapy was initiated. By the second day of hospitalization, insulin glargine was increased to 8 units (0-0-0-8 U) while the sliding scale therapy was continued, yet there was no significant improvement in pre-prandial blood glucose levels compared to day 1.

By day 3, the sliding scale was discontinued, and regular pre-prandial injection of insulin aspart commenced at a dose of 12-8-10 U, along with an increase in insulin glargine to 12 units (0-0-0-12 U).

On day 4, the dosage of insulin aspart was modified to 12-6-6 U, and insulin glargine was further increased to 16 units (0-0-0-16 U), yet the patient’s pre-prandial glucose levels persisted high (331–435 mg/dL).

Insulin aspart was then increased to 12-6-10 U at day 5 and then to 12-8-12 U at day 6. At day 6, insulin glargine was also increased to 18 units (0-0-0-18 U). However, the patient’s glucose level remained high and was difficult to control (pre-prandial glucose levels being 212–269 mg/dL). Despite the possibility of increasing the basal insulin injection, the patient expressed reservations about dose escalation, and therefore, after checking that the urine ketone bodies were negative, SGLT2 inhibitor, dapagliflozin (5 mg), was added on top of the regular insulin therapy. Following the addition of dapagliflozin, the patient’s blood glucose level stabilized, maintaining pre-prandial blood glucose levels around 135 mg/dL.

Although HbA1c level was recorded at 8.6% upon discharge, it demonstrated gradual improvement over time. Dapagliflozin was continued, and in the 2-month follow-up after discharge, the HbA1c level was 8.3%. The dosage of insulin glargine was reduced to 16 units (0-0-0-16), while the insulin aspart dosage was increased to 18-12-14 U, based on self-monitoring blood glucose (SMBG) results. At the 4-month follow-up, the HbA1c level further decreased to 8.2% with insulin glargine reduced to 10 units (0-0-0-10 U) and insulin aspart reduced to 10-4-8 U. By the 6-month mark, the HbA1c level stabilized at 7.6% with the continued administration of dapagliflozin, insulin aspart (10-8-8 U), and insulin glargine (0-0-0-8 U).

## Methods

The English language written case reports published before June 2023 were searched using PubMed with the terms “nivolumab,” “ipilimumab,” “diabetes,” “diabetes mellitus,” and “PD-1 inhibitor.” A total of 48 cases from 41 reports were obtained (18–58). The cases with no history of diabetes and using nivolumab, ipilimumab, or both were extracted. The information, such as age, sex, tumor type, plasma glucose level, HbA1c, islet autoantibodies, and treatment for hyperglycemia, was extracted. All the values are described as medium (IQR: interquartile range). Statistical analysis was conducted using the Mann–Whitney *U*-test. A *p*-value of <0.05 was considered as significant difference.

The informed consent was obtained from the patient for the case report.

## Results

The patients collected in this systematic review comprised a female/male ratio of 23/25, with an age of 64.5 (49–73.5) years old. Tumor types consisted of melanoma in 37% (18/48), non-small-cell lung cancer in 27% (13/48), renal cell carcinoma in 14% (7/48), and other types in 21% (10/48).

All patients received nivolumab, with 11 patients also receiving ipilimumab. The plasma glucose level for all the patients was 571 (384–743) mg/dL, and the HbA1c level was 7.7 (6.7–8.8) %. The plasma glucose levels between nivolumab-treated patients and nivolumab + ipilimumab-treated patients revealed no significant difference [539 (390–739) mg/dL vs. 603 (355–763) mg/dL, respectively]. However, HbA1c level showed a significantly lower level in nivolumab + ipilimumab-treated patients [8.0 (7.1–9.1) % vs. 6.9 (6.6–7.6) %: *p* = 0.048]. The serum C-peptide level was not reported in all cases, but 29 cases reported its level, and 14 cases had levels less than 0.1 ng/mL. Glutamate decarboxylase antibody (GADA) was present in 27.8% (10/36) of cases, while islet cell antibody was positive in 14.3% (4/28) of cases.

All patients were treated with insulin injection therapy for hyperglycemia with three patients also receiving additional oral anti-diabetic treatments. The first patient received an additional DPP4 inhibitor treatment (specific drug not described), the second patient received sitagliptin + acarbose, and the third patient received metformin and acarbose. There were no reports of patients receiving SGLT2 inhibitor treatment.

## Discussion

The prevalence of adverse events in endocrine organs due to CPIs has been reported to range from 4 to 30% (59, 60). Among these adverse events, CPI-triggered diabetes is exceptionally rare, accounting for less than 1% of cases (7). CPI-triggered diabetes exhibits features characterized by the sudden onset of high blood glucose levels and relatively low HbA1c often accompanied by ketoacidosis (8, 10, 59). Our present case aligns with these features as it demonstrated both a high blood glucose level and relatively low HbA1c.

In animal studies, non-obese diabetic mice showed rapid onset of diabetes by blocking the PD-1 and PD-L1 pathway (61–68).

CPI-triggered diabetes shares common characteristics with type 1 diabetes. Similar to type 1 diabetes, CPI’s adverse effects target insulin-producing pancreatic beta cells, leading to insulin deficiency and subsequent hyperglycemia (8).

Most CPIs that are known to trigger diabetes belong to the PD-1 inhibitors, and as far as we know, there have been no reports of diabetes triggered solely by CTLA-4 inhibitors (8, 12). PD-L1 expression is reported to be increased in type 1 diabetic patients compared to type 2 diabetic patients or healthy controls (20). Interestingly, to date, the expression of CTLA-4 in pancreatic islets has not been reported. This explains why CTLA-4 inhibitors alone do not induce adverse diabetic events. However, combination therapy of PD-1 inhibitor and CTLA-4 inhibitor has been reported to increase the risk of developing diabetes (69), which is consistent with the case in the present report. Although CTLA-4 may not be expressed in pancreatic beta cells, it is considered to play an important role in glucose regulation. This was further confirmed by studies showing that individuals with polymorphism in PD-1 and CTLA-4 genes are more susceptible to autoimmune disorders, including type 1 diabetes (70–74).

CPI-triggered diabetes is similar to type 1 diabetes in that they have a common feature of insulin deficiency. Therefore, as in the case of type 1 diabetes, SGLT2 inhibitors may be potentially useful for the treatment of CPI-triggered diabetes. However, it is important to point out that our present case had moderate renal impairment and SGLT2 inhibitors are not generally recommended to be used in patients with severe renal impairment. Therefore, our present case was carefully taken care for the development of further renal impairment, and our case is not a typical case for using SGLT2 inhibitor and it is a limitation of this report.

The prevalence of ketoacidosis in type 1 diabetes patients is reported as high as 4–6% when SGLT2 inhibitors are used in combination with insulin, although it is rare in type 2 diabetes patients. The FDA adverse event report system found a 7-fold higher risk of acidosis with SGLT2 inhibitors compared to dipeptidyl peptidase 4 inhibitor therapy in type 2 diabetes patients (16, 75–77).

The underlying mechanism of ketoacidosis in patients treated with SGLT2 inhibitors is induced by glucose loss, leading to lipolysis of fat mass and a decrease in the insulin/glucagon ratio (17, 69). This leads to an increase in acetyl-CoA production from fatty acids and β-oxidation, ultimately inducing ketoacid production. The risk of SGLT2 inhibitor-associated ketoacidosis increases when insulin deficiency becomes acutely pronounced or with a sudden restriction of carbohydrate availability (17, 78). In the present case, an SGLT2 inhibitor was administered only after the acute phase of CPI-triggered diabetes was confirmed to be relieved with urine ketone body negative and food intake becoming normal. So far, the present report is the first successful case of using SGLT2 inhibitor in addition to insulin in CPI-triggered diabetes.

Since a high blood glucose level may have a negative effect on cancer treatment, maintaining good glycemic control is important in cancer patients. As we have shown in the present case, with close monitoring and appropriate adjustment, SGLT2 inhibitors may be an effective solution in CPI-triggered diabetes when urine ketone bodies are negative, and long-term glycemic control is not achieved by insulin therapy alone.

## Conclusion

In summary, CPIs are widely used for cancer treatment, and as a result, the incidence of CPI-triggered diabetes is on the rise. Insulin therapy is typically considered the best approach for managing CPI-triggered diabetes. However, our present case highlights that when insulin therapy fails to produce the desired effect, the addition of SGLT2 inhibitor can effectively achieve optimal glycemic control. Although SGLT2 inhibitors have been associated with adverse effects on ketoacidosis, recent studies suggest that the occurrence of ketoacidosis is relatively rare. Given the pathological mechanism of CPI-triggered diabetes, SGLT2 inhibitors could represent an effective option provided they are administered with diligent ketoacidosis monitoring.

## Data availability statement

The original contributions presented in the study are included in the article/supplementary material, further inquiries can be directed to the corresponding author.

## Ethics statement

Written informed consent was obtained from the participant/patient(s) for the publication of this case report.

## Author contributions

MF: Data curation, Formal analysis, Investigation, Resources, Writing – original draft. MS: Formal analysis, Investigation, Resources, Writing – original draft. TO: Data curation, Formal analysis, Writing – review & editing. YM: Conceptualization, Data curation, Investigation, Writing – review & editing. KS: Project administration, Supervision, Writing – original draft, Writing – review & editing.
